# Management of Hypervascular Retained Products of Conception With Massive Bleeding

**DOI:** 10.7759/cureus.45952

**Published:** 2023-09-25

**Authors:** Takahiro Shimada, Yu Wakimoto, Mariko Kamihigashi, Hiroyuki Tanaka, Hiroaki Shibahara

**Affiliations:** 1 Department of Obstetrics and Gynecology, Hyogo Medical University Hospital, Hyogo, JPN

**Keywords:** placental remnants, uterine artery pseudoaneurysms, hypervascular rpoc, post abortion bleeding, massive postpartum hemorrhage

## Abstract

Retained products of conception (RPOC) could be a factor for massive postpartum hemorrhage; however, a management protocol is yet to be established. Performing a surgical intervention is controversial due to the potential for natural healing. Herein, we report the management of a hypervascular RPOC case with massive bleeding. Abortion was performed in a 40-year-old patient with gravida 2 and para 0, at 20 weeks and five days of gestation following the detection of Down’s syndrome on prenatal screening. Post-delivery transvaginal ultrasonography identified an intrauterine mass measuring 4cm × 5cm × 5cm. The patient was then followed up in the outpatient department. One month after the abortion, the patient developed abnormal vaginal bleeding. Transvaginal ultrasonography revealed a hypervascular myometrial RPOC with turbulent flow. Although the bleeding stopped upon her admission to our hospital, the patient developed recurrent abnormal vaginal bleeding after nine days of hospitalization, which resulted in a hemoglobin level drop to 5.9 g/dL. CT and MRI scan findings raised the suspicion of hypervascular RPOC or uterine artery pseudoaneurysm. Uterine artery embolization was performed, leading to diminished vascularity in the RPOC, which was confirmed through color Doppler ultrasonography. The remnant placenta was successfully resected hysteroscopically, and a subsequent transvaginal ultrasonography showed a decrease in blood flow. In conclusion, hypervascular RPOC, previously reported as uterine artery pseudoaneurysms, should be considered when detecting hypervascular myometrial lesions in postpartum ultrasonography. Hypervascular RPOC with hemorrhage might benefit from hysteroscopic resection after achieving hemostasis with uterine artery embolization. This case report highlights the potential risks of awaiting spontaneous resolution in large RPOC and suggests that timely surgical intervention is both effective and essential.

## Introduction

Retained products of conception (RPOC) refer to undelivered pregnancy tissue, such as the placenta and amniotic sac, in the uterine cavity following miscarriage or delivery [1,2]. The pathogenesis of RPOC is reportedly placental remnants, migration of trophoblastic tissue into the deep decidual veins, or coagulation of the rough placental abruption surface of the postpartum uterus caused by atonic uterine contractions [3]. Atonic uterine contractions prevent spiral uterine artery constriction and decrease bleeding; thus, blood flow cannot be stopped via vessel regression [3]. The trophoblast-remodeled uterine vascularity causes hypervascular RPOC. The hypervascular RPOC is frequently detected on color Doppler ultrasound [3] and is described as variable vascularity of the remainder villi [3]. Although hypervascular RPOC is a factor in massive postpartum hemorrhage, management strategies have not been established [2]. It is necessary to decide whether a follow-up with a wait-and-watch strategy or therapeutic interventions, such as dilatation and evacuation (D&E), hysteroscopic transcervical resection (TCR), uterine artery embolization (UAE), or total hysterectomy, are required [1]. However, pseudoaneurysms are caused by arterial injuries due to invasive procedures such as cesarean section or intrauterine scraping, as well as non-invasive procedures such as miscarriage or delivery [4,5]. Additionally, hypervascular RPOC needs to be differentiated from the pseudoaneurysm of the uterine artery [4,5]. Hypervascular RPOC with heavy bleeding is curable by total hysterectomy [5]; however, it leads to fertility loss. Herein, we describe a case of successful management of hypervascular RPOC with massive bleeding by using UAE followed by TCR. This article was previously presented as a meeting abstract at the 75th Annual Congress of the Japan Society of Obstetrics and Gynecology (JSOG 2023) on May 12, 2023.

## Case presentation

A 40-year-old woman, with a history of two pregnancies and 0 delivery, conceived spontaneously. Owing to her age, she underwent non-invasive prenatal genetic testing at our hospital. Amniotic fluid analysis revealed that the fetus had Down’s syndrome 46XY, i(21)(q10); thus, it was terminated at 20 weeks and five days of gestation. We also offered genetic counseling to discuss the possibility that one of the individuals in the couple might be a carrier. However, they expressed no intention of planning for a future pregnancy, and they declined to undergo genetic testing.

**Figure 1:**
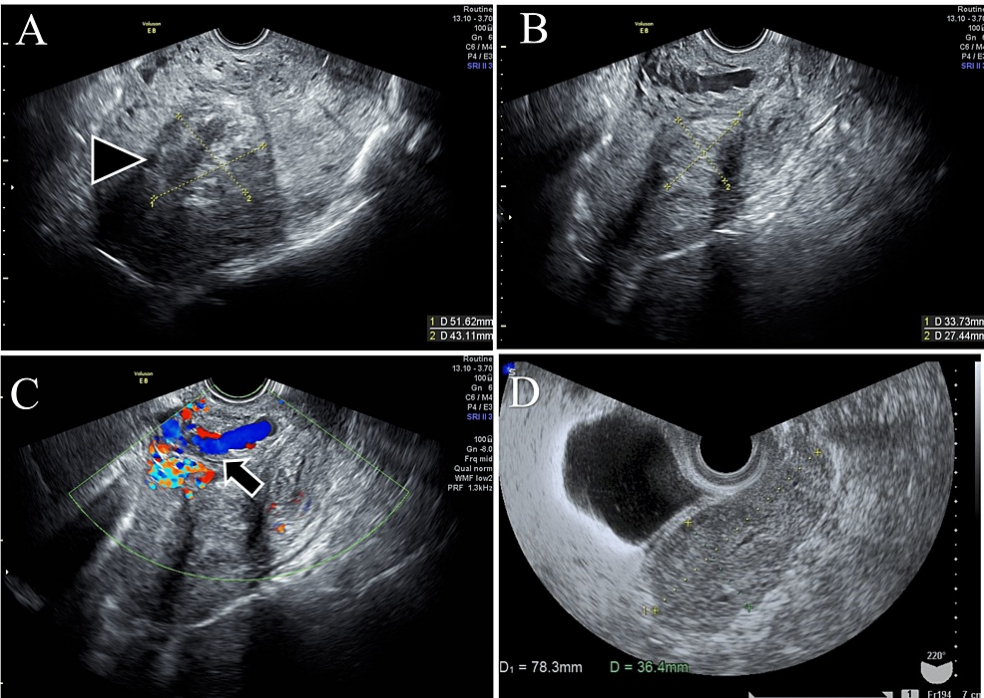
Transvaginal ultrasonography image A) Transvaginal scan on day 1 postpartum showing intrauterine mass (head arrow). B) Transvaginal ultrasonography image on postpartum day 35 showing an echogenic area in the muscular layer of the anterior wall of the uterus. C) Transvaginal color Doppler ultrasonography confirmed angiogenesis, with abundant blood flow in the same area (arrow). D) Transvaginal ultrasound image obtained 78 days after transcervial resection: there were no obvious pooling in the uterine cavity, and the angiogenesis shown in Figure C had disappeared.

After the second trimester abortion, she lost 1,876 ml of blood owing to atonic bleeding, inducing hypovolemic shock and a drop in hemoglobin level to 5.4 g/dL. Six units of red blood cells and four units of fresh frozen plasma (FFP) were transfused. The hemoglobin levels improved to 8.3 g/dL. Transvaginal ultrasound on the first postpartum day showed an intrauterine mass (Figure 1A). The patient was discharged on postpartum day 5 without any abnormal bleeding or obvious placental remnants on ultrasonography (Figure 1).

Approximately one month after discharge from our hospital, on postpartum day 35, the patient was transported by ambulance and admitted to our hospital for massive genital bleeding. At the time of arrival, the hemoglobin level was 8.3 g/dL, and there was no active genital bleeding. The patient was followed up without any surgical intervention. Transvaginal color Doppler imaging revealed angiogenesis in the muscular layer of the anterior uterine wall (Figures 1B-1C).

**Figure 2:**
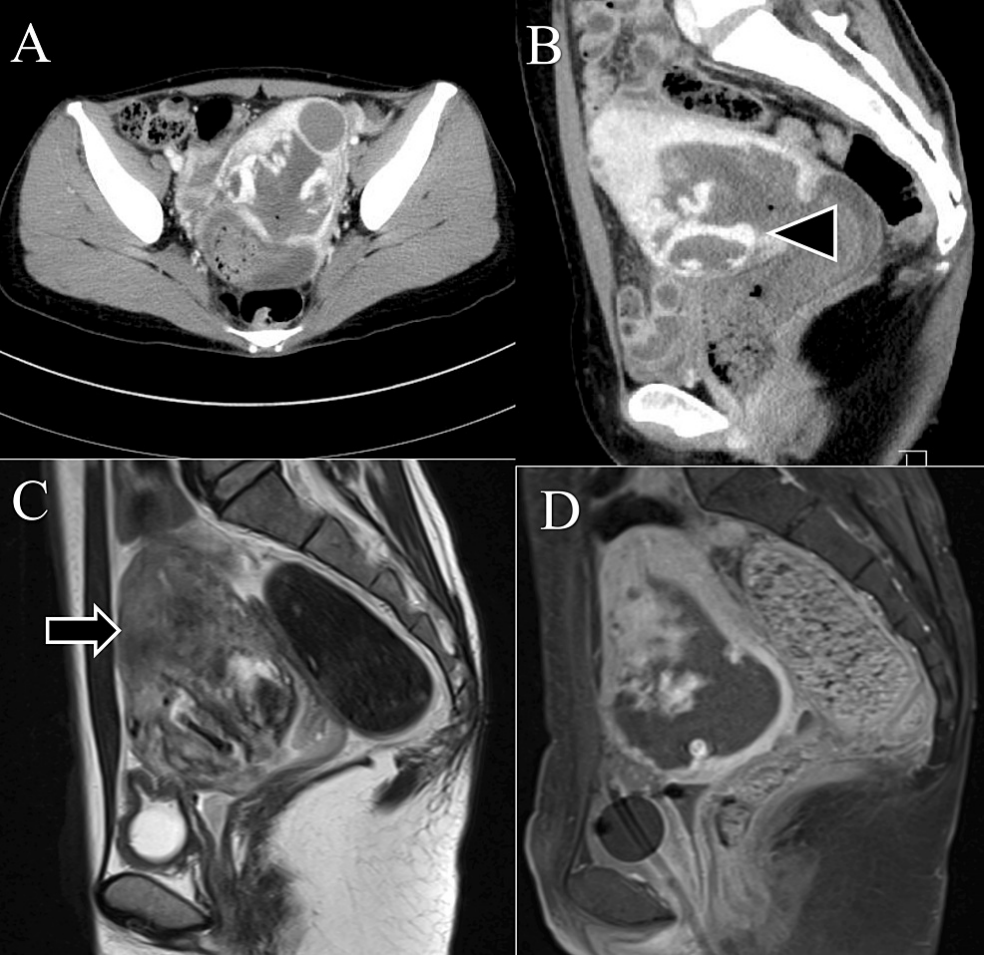
Contrast-enhanced CT and MRI A) and B) show the contrast-enhanced computed tomography images performed on postpartum day 44. A hypervascular lesion was observed within the myometrium(head arrow), suggesting the possibility of a pseudoaneurysm or hypervascular RPOC. C) and D) show the magnetic resonance images on postpartum day 44. Large remnants were visualized in the uterine cavity, suggesting the possibility of RPOC. The junctional zone had disappeared(arrow), and vascular infiltration into the myometrium was confirmed (C and D).

The patient was discharged on postpartum day 44. However, the same night, she was readmitted due to massive bleeding. Four units of red blood cells were transfused for a hemoglobin level of 5.9 g/dL. Contrast-enhanced CT and MRI scans were performed (Figure 2). Contrast-enhanced CT revealed an arteriovenous shunt, and a uterine pseudoaneurysm was suspected. Contrast-enhanced MRI revealed contents suggestive of intrauterine placental remnants with marked blood flow.

**Figure 3:**
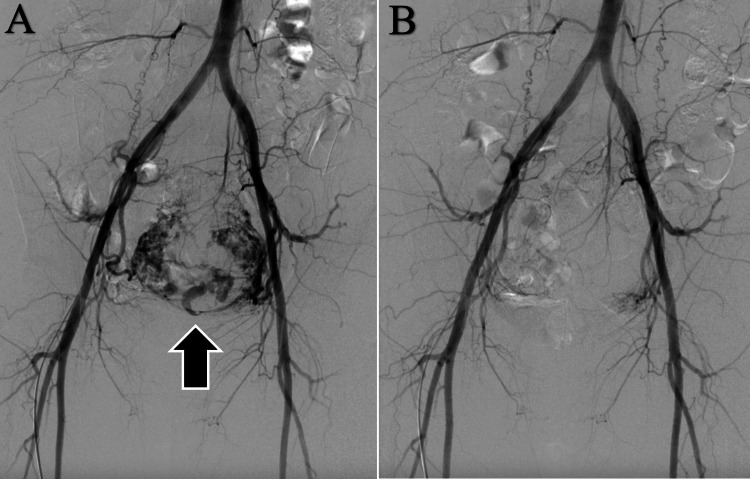
Angiographic images before and after uterine artery embolization. A) Abundant blood flow was observed in the myometrial areas of vascularization(arrow), suggesting hypervascular RPOC. B) After embolization, > 90% of the abundant blood flow in the myometrial areas of vascularization was occluded.

Uterine artery embolization (UAE) was performed on the same day. Angiography during UAE showed neovascularization of the intrauterine placental remnants. More than 90% of the feeding vessels were embolized, and hemostasis was achieved (Figure 3).

Transcervical resection (TCR) was performed four days after UAE. A hysteroscopic examination confirmed the presence of an endometrial mass, which was successfully removed without resistance using placental forceps. The mass measured approximately 55 mm × 30 mm; histopathological examination revealed degenerative placental tissue. After TCR, the genital bleeding improved, ultrasonography showed blood flow reduction, and the patient was discharged on postoperative day 11. Transvaginal ultrasonography on postoperative day 78 confirmed the disappearance of intrauterine vascularization (Figure 1D). Hysteroscopy on postoperative day 181 confirmed the absence of residual content in the uterine cavity. Menstruation resumed on day 69 post-UAE. This patient agreed to our treatment and we obtained written informed consent from her.

## Discussion

Herein, we report a case of hypervascular RPOC with massive hemorrhage during the postpartum period. We initially opted for a wait-and-watch approach. Because uterine cavity bleeding occurred again, we performed UAE followed by TCR. However, there is currently no established management strategy for the treatment of RPOC [2,3]. Standby therapies including TCR, intrauterine curettage, UAE, and total hysterectomy are usually done to manage. Approximately 80% of patients with post-miscarriage RPOC achieve remission after standby therapy [2,3]. However, in cases of heavy bleeding at the time of miscarriage or in hypervascular RPOC, surgical interventions are required [3]. Wait-and-watch therapy is a good strategy in RPOC cases with minimal bleeding; a spontaneous decrease in blood flow during follow-up has been reported [6,7]. However, sudden massive genital bleeding could occur during the standby treatment; thus, follow-up at a medical institution capable of blood transfusions, UAE, and emergency total hysterectomy is needed. Radical surgical interventions include TCR, endometrial curettage, and total hysterectomy. Takahashi et al. reported that surgical intervention is necessary in cases with RPOC > 4 cm or with hypervascularity [2]. Furthermore, patients who undergo UAE or total hysterectomy for RPOC have significantly higher volumes of blood loss and transfusions during delivery and higher incidences of hypervascular RPOC and RPOCs > 4 cm in size than those who did not undergo UAE or total hysterectomy [1]. These findings could be useful as a criterion for determining surgical intervention needs. Our case fulfilled all these criteria, suggesting that surgical intervention may be more appropriate than wait-and-watch therapy.

We performed UAE, followed by TCR, for the rebleeding. Intrauterine curettage of the endometrium was performed blindly. However, TCR allows for RPOC resection under direct vision using a camera. TCR has a lower intrauterine adhesion rate and higher post-operative pregnancy rate than intrauterine curettage [8]. However, in cases of massive hemorrhage and hypervascular RPOC, the visual field is poor, making it difficult to remove the RPOC via TCR. We believe that preoperative UAE for hypervascular RPOC is effective because it obtains homeostasis, enabling invasive procedures by TCR. UAE is one of the most useful options for treating uterine pseudoaneurysms, which must be differentiated from hypervascular RPOC. However, treatment of uterine pseudoaneurysms in the postpartum period should be determined on a case-by-case basis because small uterine pseudoaneurysms spontaneously resolve [9]. However, if it is difficult to differentiate between hypervascular RPOC and uterine pseudoaneurysms, UAE should be considered. Furthermore, in cases of massive genital bleeding or hypervascular RPOC on imaging findings, UAE should be considered to reduce uterine blood flow followed by wait-and-watch therapy or surgical intervention. Therefore, the potential effect of UAE on post-operative ovarian function and pregnancy should be considered in future studies. The use of a permanent spherical embolization material (embosphere) that reduces ovarian function, should be avoided in UAE; gelatin sponge materials should be used. Doumouchtsis et al. found that menstruation restarted within 6 months postpartum in 91% of women after UAE, and 78% of women who wanted to conceive were pregnant [10].

The incidence of RPOC decreases with births after 22 weeks of gestation, with an incidence of 1% at term [1]. The incidence increases with miscarriage or abortion at < 22 weeks of gestation (6.7-29%) [1]. Risk factors for RPOC development include placental abnormalities such as placenta accreta, accessory placenta, and lobed placenta; uterine surgery causing endometrial damage; and abnormal uterine cavity morphology [11,12]. The presence of trophoblastic tissue on histopathology is definitive of RPOC [5]. Our patient had placenta accreta and other risk factors for RPOC. However, in clinical practice, a clinical diagnosis must be made before a histological diagnosis to determine the treatment strategy. Initially, the wait-and-watch approach with ultrasonographic follow-up was used in our patient; however, because of the massive hemorrhage, an MRI scan was performed. Shiina et al. determined characteristic MRI findings indicative of hypervascular RPOC [5], three of which were found in our case: the presence of a remnant, the break of the junctional zone in contact with the remnant, and vascularization/flow voids infiltrating into the myometrium from the broken junctional zone [5]. Hypervascular RPOC involves the uterine cavity mass and can be distinguished from uterine artery pseudoaneurysms, which primarily involve only the myometrium. However, the diagnosis of hypervascular RPOC remains difficult owing to its similarities with uterine artery pseudoaneurysms in terms of clinical course and imaging findings [4,5]. Furthermore, the possibility of overlap between the two should be considered. Uterine angiogenesis, which spontaneously regresses after miscarriage or delivery, reportedly causes uterine artery pseudoaneurysms; however, these reports may include hypervascular RPOC [5]. Therefore, if an area of abundant blood flow is found in the myometrium on postpartum ultrasonography, postpartum should be managed with hypervascular RPOC in mind and differentiated from a pseudoaneurysm of the uterine artery.

## Conclusions

Hypervascular RPOC, previously reported as uterine artery pseudoaneurysms, should be taken into consideration when detecting hypervascular myometrial lesions in postpartum ultrasonography. Hypervascular RPOC with hemorrhage can be managed with TCR following hemostasis achieved through UAE. This case report underscores the potential risk of massive bleeding when waiting for spontaneous resolution in large RPOC and suggests that prompt surgical intervention could be both effective and essential. Patients must be adequately informed about their medical condition and available treatment options; subsequently, the treatment plan should be tailored to their preferences and overall health.

## References

[1] Takahashi H, Tanaka H, Osuga Y (2022). Retained products of conception (RPOC) following delivery without placenta previa: which patients with RPOC show postpartum hemorrhage?. Placenta.

[2] Takahashi H, Ohhashi M, Baba Y (2019). Conservative management of retained products of conception in the normal placental position: a retrospective observational study. Eur J Obstet Gynecol Reprod Biol.

[3] Wada Y, Takahashi H, Suzuki H (2021). Expectant management of retained products of conception following abortion: A retrospective cohort study. Eur J Obstet Gynecol Reprod Biol.

[4] Iraha Y, Okada M, Toguchi M (2018). Multimodality imaging in secondary postpartum or postabortion hemorrhage: retained products of conception and related conditions. Jpn J Radiol.

[5] Shiina Y, Itagaki T, Ohtake H (2018). Hypervascular retained product of conception: characteristic magnetic resonance imaging and possible relationship to placental polyp and pseudoaneurysm. J Obstet Gynaecol Res.

[6] Mori M, Iwase A, Osuka S (2016). Choosing the optimal therapeutic strategy for placental polyps using power Doppler color scoring: Transarterial embolization followed by hysteroscopic resection or expectant management?. Taiwan J Obstet Gynecol.

[7] Timmermans S, van Hof AC, Duvekot JJ (2007). Conservative management of abnormally invasive placentation. Obstet Gynecol Surv.

[8] Ben-Ami I, Melcer Y, Smorgick N, Schneider D, Pansky M, Halperin R (2014). A comparison of reproductive outcomes following hysteroscopic management versus dilatation and curettage of retained products of conception. Int J Gynaecol Obstet.

[9] Takahashi H, Baba Y, Usui R, Ohkuchi A, Kijima S, Matsubara S (2016). Spontaneous resolution of post-delivery or post-abortion uterine artery pseudoaneurysm: a report of three cases. J Obstet Gynaecol Res.

[10] Doumouchtsis SK, Nikolopoulos K, Talaulikar V, Krishna A, Arulkumaran S (2014). Menstrual and fertility outcomes following the surgical management of postpartum haemorrhage: a systematic review. BJOG.

[11] Baba T, Endo T, Ikeda K (2013). Assisted reproductive technique increases the risk of placental polyp. Gynecol Endocrinol.

[12] Miyahara Y, Makihara N, Yamasaki Y, Ebina Y, Deguchi M, Yamada H (2014). In vitro fertilization-embryo transfer pregnancy was a risk factor for hemorrhagic shock in women with placental polyp. Gynecol Endocrinol.

